# Development and validation of a novel model for predicting the survival of bladder cancer based on ferroptosis-related genes

**DOI:** 10.18632/aging.204385

**Published:** 2022-11-17

**Authors:** Lianjun Li, Leizuo Zhao, Bin Li, Tengteng Wang, Weiting Kang, Zilian Cui, Dongjian Liu, Da Gu

**Affiliations:** 1 Department of Urology, Shandong Provincial Hospital Affiliated to Shandong First Medical University, Jinan, Shandong 250021, China; 2 Department of Urology, Dongying People’s Hospital, Dongying, Shandong 257091, China; 3 Department of Plastic Surgery, Central Hospital Affiliated to Shandong First Medical University, Jinan, Shandong 250013, China

**Keywords:** bladder cancer, ferroptosis, nomogram

## Abstract

The role of ferroptosis, a new form of cell death, in bladder cancer (BC) has not been sufficiently studied. This study aimed to establish a prognostic prediction model for BC patients based on the expression profile of ferroptosis-related genes (FRG). The expression profiles of BC samples with clinical information were obtained from The Cancer Genome Atlas (TCGA) and Gene Expression Omnibus (GEO). A total of 80 differentially expressed genes (DEGs) related to FRG were identified among which 37 DEGs were found to have a prognostic value. Eleven genetic markers including SLC2A12, CDO1, JDP2, MAFG, CAPG, RRM2, SLC2A3, SLC3A2, VDAC2, GCH1, and ANGPTL7 were identified through the LASSO regression analysis. The ROC curve analysis showed that the AUC was 0.702, 0.664, and 0.655 for the 1-year, 3-year, and 5-year survival outcomes, respectively. The prediction performance was verified in the TCGA-testing set and external set GSE13507. Multivariate Cox proportional hazards analysis showed that the risk score was an independent prognostic predictor. Moreover, we found differences in gene mutation, gene expression, and immune cell infiltration between the high and low-risk groups of BC patients. Finally, a nomogram was constructed by integrating clinical features and FRG signatures to predict the survival outcomes of BC patients. In addition, the differential expression of FRG mRNA and protein was verified through PCR and HPA online site. The characteristics of 11 FRG genes were examined and a prognostic nomogram for predicting the overall survival of BC was established.

## INTRODUCTION

Bladder cancer has the characteristics of high morbidity and mortality [1]. In 2022, 81,180 new BC cases and 17,100 BC deaths will be detected in the U.S. [2]. It was reported that about 75% of BC patients had non-muscle invasive bladder cancer (NMIBC), and about 25% had muscle invasive bladder cancer (MIBC) or metastatic BC at first diagnosis [3, 4]. Non-muscle invasive bladder cancer has the highest recurrence rates in the range of 50% to 70% and progression rates ranging from 10% to 30% [5]. The annual cost of care for NMIBC patients has also increased over time, especially among those with recurrent disease [6]. When NMIBC progresses to MIBC, the five-year overall survival (OS) rate is 60%–70% [7, 8]. Therefore, it is particularly important to construct prognostic models to predict bladder cancer progression and evaluate treatment outcomes.

Ferroptosis is a novel form of programmed cell death characterized by elevated intracellular iron content and lipid reactive oxygen species (ROS) [9]. The occurrence of ferroptosis is affected by many biological processes, such as the metabolism of amino acids, iron and polyunsaturated fatty acids, and the biosynthesis of glutathione, phospholipid, NADPH and coenzyme Q10. A growing body of research found that ferroptosis was associated with a variety of diseases including cancers, blood diseases, immune system diseases, brain and neurodegenerative diseases, and heart diseases [10]. Accumulating evidence suggests that ferroptosis may be triggered by various cancer treatments, especially in the treatments of invasive malignancies that are resistant to traditional therapies [11]. Tumor progression can be inhibited by inducing ferroptosis, so ferroptosis can be used as an effective means of treating cancer. Therefore, it is necessary to analyze the expression and prognostic value of FRG genes in BC to provide a theoretical basis for the construction of a prognostic risk model and cancer treatments.

We used the TCGA and GSE13507 datasets to establish and validate 11 FRG gene signatures for BC patients based on FRG genes in the FerrDb. The BC patients were divided into high-risk and low-risk groups based on 11 FRG genes. Group differences in terms of gene mutation, differential expression, and immune cell infiltration levels, were investigated to elucidate their underlying pathogenic mechanisms. This study identified a prognostic model based on 11 FRG gene signatures for predicting the risk and prognosis of BC patients in order to guide clinical treatment.

## MATERIALS AND METHODS

### Data download and preprocessing

Fragments per Kilobase of transcript per Million mapped reads (FPKM) standardized RNA sequencing datasets and clinical data were downloaded from the TCGA database. The TCGA-BLCA cohort included 411 and 19 bladder cancer tissues and non-tumor tissues, respectively. Additionally, we downloaded gene expression data and clinical information for the GSE13507 dataset from the GEO database (https://www.ncbi.nlm.nih.gov/geo/). The GSE13507 dataset includes a total of 165 primary tumors, 9 normal tissues, 58 control tissues and 23 recurrent tissues [12, 13]. Also, masked somatic mutation data of bladder cancer patients were downloaded from TCGA.

### Differential expression and prognostic analysis

The R language “limma” package was used to analyze differences expression of genes between BC and adjacent tissues [14]. The screening standard of differential genes was that the absolute value of the difference fold is greater than 1, and the adjusted p value is less than 0.05. Patients with a >30 days survival time were screened for prognostic analysis using the Cox proportional hazards regression model and Survival analysis (Log-Rank test). Adjusted *P* < 0.05 was used to identify prognosis-related differential FRG. 37 prognosis-related FRG genes were considered as the initial candidate FRGs.

### Construction of a prognostic model for FRG genes

Bladder cancer patients were randomly divided into the TCGA-training set and the TCGA-testing set in the TCGA dataset. The ratio of the two cohorts was 7:3 to ensure that the proportion of BC stages was the same in both groups. Based on the initial candidate FRGs, a prognostic model was constructed in the training set using LASSO regression analysis and a risk score was calculated for each patient with BC. Subsequently, the Youden index was calculated from the 5-year survival ROC curve. The optimal threshold was set as the highest Youden index at which the threshold was far from the change line, with high sensitivity and specificity. Finally, the optimal threshold was determined as the cutoff value, and bladder cancer patients were divided into high-risk and low-risk groups with cutoff values. Survival analysis was performed using the R software “survival” package to calculate prognostic differences in different risk groups. Subsequently, we calculated the area under the curve (AUC) at 1 year, 3 year, and 5 years to assess the discriminative power. In the TCGA-testing set and GSE13507, the expression values and coefficients of the same genes were used again to calculate the risk scores of BC patients, and the cutoff value was used to classify them into different risk groups, thereby verifying the validity of the prognostic model.

### Prognostic value of risk score was assessed using Cox proportional hazards

Clinical characteristics of BC patients, including age, gender, pathological TNM stage, pathological grade, survival time and status, were collected from TCGA database. We assessed the prognostic value of risk scores and clinical data using univariate and multivariate Cox proportional hazards.

### The human protein atlas was used to assess protein expression levels

The Human Protein Atlas (HPA, https://www.proteinatlas.org/) was initiated in Sweden, and the database uses a variety of omics techniques to detect human protein expression levels at the cell, tissue and organ levels [15]. We used HPA to assess the differential expression of ferroptosis-related proteins in bladder cancer tissues and normal tissues.

### Assessing immune cell infiltration in bladder cancer tissues

We used the analytical tool R package “CIBERSORT” developed by Newman et al. to analyze the degree of immune cell infiltration in bladder cancer tissues [16]. CIBERSORT can use transcriptome sequencing results to assess the level of immune cell infiltration, and calculate the *p*-value for each sample, and select samples with *p* < 0.05 for subsequent analysis.

### Function enrichment analysis

Differential genes in different groups were screened, and GO enrichment and KEGG analysis were performed on the differential genes using the R package ‘clusterprofiler’ [17]. Adjusted *p* < 0.05 for GO enrichment or KEGG pathway was considered statistically significant.

### Collection of clinical specimens

Tumor tissue and para-cancerous tissue from 15 patients who underwent radical resection of bladder cancer at the Shandong Provincial Hospital Affiliated to Shandong First Medical University between 2020 and 2021. Inclusion criteria were bladder cancer determined by pathological examination. The Ethics Committee of the Shandong Provincial Hospital Affiliated to Shandong First Medical University approved the study, and we obtained signed informed consent from patients.

### RNA isolation and real-time PCR

Tissues of appropriate size were taken, and total mRNA was extracted from the tissues using TRIzol reagent (Invitrogen), and the concentration of mRNA was measured. According to the manufacturer’s instructions, mRNA was reverse transcribed to cDNA using PrimeScript^™^ RT Master Mix (Takara). Subsequently, primers for FRGs were designed and synthesized, and their expression levels were detected by real-time PCR using SYBR-Green kit (Takara). The primer sequences are shown in Supplementary Table 1.

### Statistical analysis

All data analyses used in this study were performed using R (R version 3.6.1). Intergroup comparisons were made using independent sample *t*-tests for measurement data, and chi-square tests or Fisher’s exact tests for enumeration data. Cumulative survival time was calculated using the Kaplan-Meier method and the differences in survival curves were analyzed using the log-rank test from the “survival” package. Cox proportional hazards was used for univariate and multivariate analysis. The ‘ggplot2’ package was used to draw boxplots and the ‘pheatmap’ package to draw heatmaps. In this study, *p* < 0.05 indicated a statistical difference.

## RESULTS

### Screening of prognostic FRG genes

**Figure 1:**
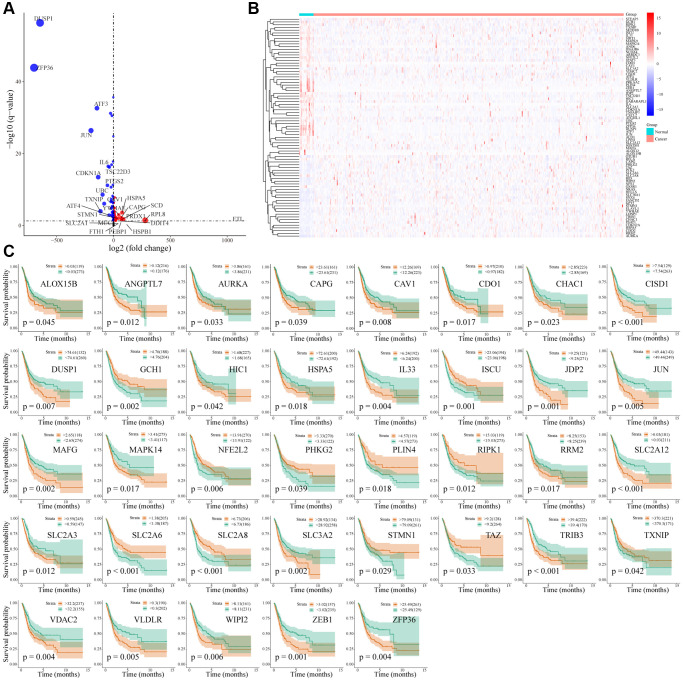
**Differential expression and prognosis of ferroptosis-related genes in bladder cancer.** (**A**) Volcano map of differentially expressed FRG in bladder cancer; (**B**) Heatmap of differentially expressed FRG in bladder cancer; (**C**) Survival analysis of 37 differentially expressed FRG in bladder cancer.

Two hundred and sixty-nine unique FRG were obtained from the FerrDb database (http://www.zhounan.org/ferrdb/). Differentially expressed FRG were screened using the TCGA data set; 430 tissues were included in TCGA (411 tumor tissues and 19 normal tissues). Differential analysis revealed 4953 differential genes (2369 upregulated and 2584 downregulated); of which 80 FRG were differentially expressed (29 FRG genes upregulated and 51 FRG genes downregulated) (Figure 1A, 1B and Supplementary Table 2).

A total of 392 patients were included in the TCGA-BLCA cohort, excluding bladder cancer patients with a survival time of less than or equal to 30 days. The Cox proportional hazards regression model and survival analysis (Log-Rank test) were used to screen out 37 FRG genes related to the prognosis of BC patients (Figure 1C). Two of the 37 FRG genes, PLIN4 and TAZ, were not in the GSE13507 data set and were excluded; therefore, 35 prognostic-related FRG genes were used for subsequent model construction.

### Construct a prognostic model for bladder cancer and internal validation

**Figure 2:**
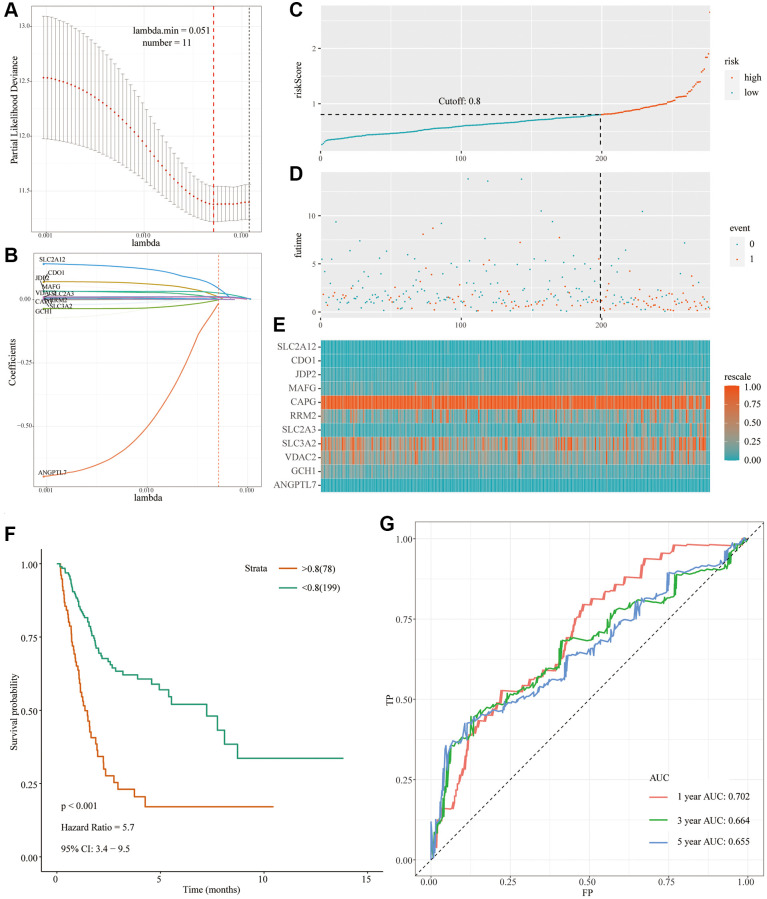
**Development of the prognostic signature based on FRGs in the TCGA-training set.** (**A** and **B**) Identification of 11 FRGs by LASSO regression analysis; (**C**) Distribution of risk scores based on FRGs in BC; (**D**) Survival status of patients in different groups; (**E**) Heatmap of the expression profiles of FRGs; (**F**) Survival analysis for the signature-defined risk groups; (**G**) Time-dependent ROC curve of the 11- FRGs prognostic signature.

In TCGA-BLCA cohort, a total of 392 patients were randomly assigned to the TCGA-training set (*n* = 277) and the TCGA-testing set (*n* = 115) according to different clinical stages in a ratio of 7:3. First, in the TCGA-training set, a prognostic model was constructed using L1 penalized Cox proportional hazards regression. 11 FRGs were screened out and the FRG index (FRGI) of related genes was calculated (Table 1, Figure 2A and 2B). Then, the FRGI was used to calculate the risk score of BC cases in the TCGA-training group. In order to determine the cutoff value, the Yunden index was first calculated based on the 5-year survival rate, and the critical value was then determined to be 0.8. Patients with bladder cancer can be classified into high risk or low risk groups based on their risk scores and cutoffs (Figure 2C and Supplementary Table 3). The prognosis of bladder cancer patients differs significantly in different risk groups. The ratio of dead patients (red dots) to surviving patients (green dots) was significantly higher in the high-risk group compared with the low-risk group (Figure 2D). As can be seen from Figure 2E, the expression levels of 11 FRGS in patients with different risk scores. Subsequently, prognostic analysis of patients in different risk groups found that OS was significantly lower in the high-risk group compared with the low-risk group (*P* < 0.001; HR = 5.7 [3.4, 9.5]) (Figure 2F). Finally, the ROC analysis (Figure 2G) showed acceptable discrimination with an AUC of 0.702 at 1 year, 0.664 at 3 years, and 0.655 at 5 years.

**Table 1:** Model information about FRGs.

**Genes**	***p*.value**	**Lasso coefficient**
SLC2A12	0.0037	0.044364646
CDO1	0.044	0.010458211
JDP2	0.035	0.001883577
MAFG	0.0022	0.026296536
CAPG	0.0089	0.001015059
RRM2	0.024	0.002248657
SLC2A3	0.00011	0.011827222
SLC3A2	0.0058	0.000836578
VDAC2	0.0021	0.008925264
GCH1	0.14	−0.000744616
ANGPTL7	0.32	−0.013603053

**Figure 3:**
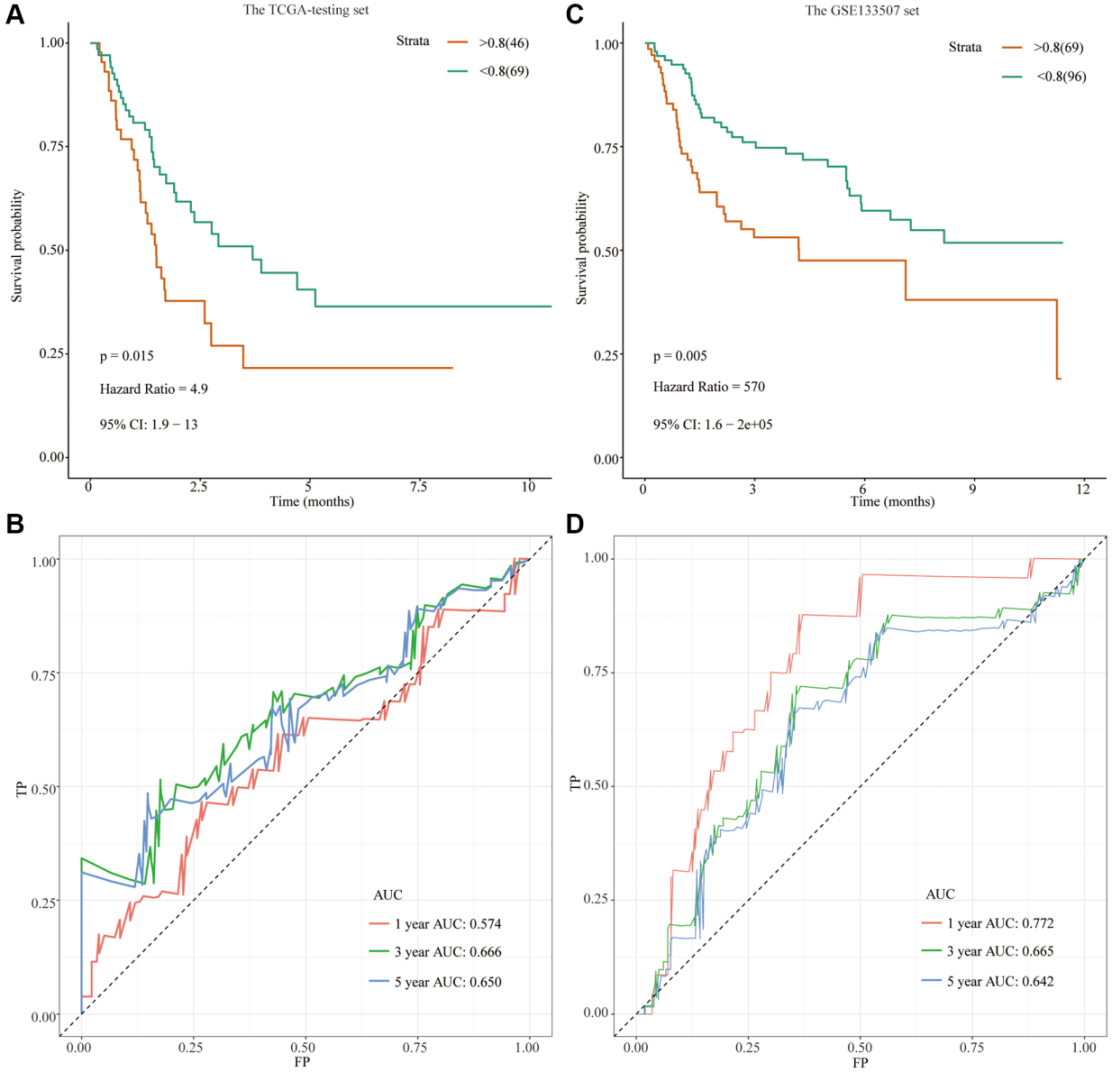
**Development of a prognostic signature based on FRGs in the TCGA-testing set and GSE13507 cohort.** (**A**) Survival analysis for the signature-defined risk groups in the TCGA-testing set; (**B**) Time-dependent ROC curve of the 11- FRGs prognostic signature in the TCGA-testing set; (**C**) Survival analysis for the signature-defined risk groups in the GSE13507 cohort; (**D**) Time-dependent ROC curve of the 11- FRGs prognostic signature in the GSE13507 cohort.

The ‘predict’ R package was used to calculate the risk scores of the TCGA-testing set, dividing bladder cancer patients into high-risk and low-risk groups according to cutoff values (Supplementary Table 3). Similarly, the clinical utility and discriminative power of 11 FRGs in the TCGA-testing set were verified. Compared with the low-risk group, OS was significantly lower in patients with bladder cancer in the high-risk group (*P* = 0.015; HR = 4.9 [1.9, 13]) (Figure 3A). As shown in Figure 3B, the ROC curve showed that the AUC value at 1 year was 0.574, the AUC value at 3 years was 0.574, and the AUC value at 5 years was 0.650, and the predicted power was acceptable.

### External validation in GSE13507 datasets

We used the ‘predict’ R package to calculate the risk scores of the GSE13507 cohort, dividing bladder cancer patients into high- and low-risk groups according to cutoff values (Supplementary Table 4). Compared with the low-risk group, OS was significantly lower in patients with bladder cancer in the high-risk group (Figure 3C). As shown in Figure 3D, the ROC curve showed that the AUC value at 1 year was 0.772, the AUC value at 3 years was 0.665, and the AUC value at 5 years was 0.642, and the predicted power was acceptable.

### Predictive power of high and low risk groups in different subgroups

Subsequently, we assessed the prognostic value of risk grouping in bladder cancer patients in different clinical subgroups (Supplementary Figure 1A–1L). The study found that that risk scores can predict the prognosis of patients in different subgroups, such as age [≥65 year (*p* < 0.0001), <65 year (*p* = 0.0017)] (Supplementary Figure 1A, 1B), sex [male (*p* < 0.0001), female (*p* = 0.00022)] (Supplementary Figure 1C, 1D), clinical stage [stage 1–2 (all risk scores were considered low), stage 2–3 (*p* < 0.0001)] (Supplementary Figure 1E, 1F), pathological stage [T1-T2 (*p* = 0.009), T2-T3 (*p* < 0.0001)] (Supplementary Figure 1G, 1H), lymph node metastasis [N0 stage (all risk scores were low), N1 stage (*p* = 0.49)] (Supplementary Figure 1I, 1J) and distant metastasis status [M0 stage (*p* < 0.0001), M1 stage (all risk scores were high)] (Supplementary Figure 1K, 1L). Then, the distribution characteristics of patients with different clinical characteristics in the high-risk and low-risk groups were assessed. There was no significant difference in the age and sex distribution of patients in the high-risk and low-risk groups (Table 2). There was 0% (0/146) of patients with distant metastasis, and 98.3% (226/230) without lymph node metastasis in the low-risk group after excluding patients with bladder cancer with lacking or with unevaluatable clinical data (Table 2). The high-risk group had 100% (122/122) of patients with lymph node metastasis, 100% (124/124) of patients with clinical stage III-IV, and 89.2% of patients with pathological stage T3-T4 (107/120) (Table 2). Therefore, based on the risk score of bladder cancer patients, their clinical-stage, pathological stage, lymphatic metastasis, and distant metastasis can be predicted.

**Table 2:** Distribution of BC patients in different risk scores and subgroups.

**Characteristic**	**Risk score**		***p*.value**
	**Low**	**High**
Age
<65 year	105	43	0.4575
≥65year	163	81	
Gender
Male	199	91	0.9537
Female	69	33	
pathologic_m
M0	146	41	6.11E-08
M1	0	10	
MX	120	73	
NA	2	0	
pathologic_n
N0	226	0	2.20E-16
N1-3	4	122	
NX	34	2	
NA	4	0	
pathologic_stage
I–II	125	0	2.2E-16
III–IV	141	124	
NA	2		
pathologic_t
T1-T2	104	13	4.84E-11
T3-T4	137	107	
TX	1	0	
NA	26	4	

### Univariate and multivariate COX regression analysis was used to verify the prognostic value of risk score

In the TCGA-BLCA cohort, the prognostic value of risk scores in bladder cancer patients was explored by univariate and multivariate Cox proportional hazards regression analysis according to the risk scores of bladder cancer patients. As shown in Table 3, in the TCGA-BLCA cohort, risk score, pathological stage, pathological stage, lymph node status and distant metastasis were found to be prognostic risk factors by univariate Cox proportional hazards regression analysis (Table 3). Multivariate analysis showed that the risk score remained an independent prognostic factor for the prognosis of bladder cancer patients after adjusting the pathological stage, pathological stage, lymph node status, and distant metastasis (HR = 3.11 [1.41–6.88], *p* = 0.005).

**Table 3:** Univariate and multivariate analyses of prognostic factors in the TCGA data set.

**Characteristic**	**Univariate**			**Multivariate**		
	**Hazard.Ratio**	**CI95**	***P*.Value**	**Hazard.Ratio**	**CI95**	***P*.Value**
RiskScore	5.54	3.54–8.68	0	3.11	1.41–6.88	0.005
Age	1	0.99–1.02	0.801	NA	NA	NA
Gender	1.03	0.73–1.44	0.875	NA	NA	NA
Pathologic T stage	1.17	1.03–1.33	0.014	1.1	0.95–1.29	0.206
Pathologic N stage	1.21	1.1–1.33	0	1.08	0.95–1.23	0.229
Pathologic M stage	1.23	1.06–1.43	0.006	1.09	0.93–1.28	0.284
Pathologic stage	1.64	1.36–1.98	0	1.18	0.9–1.56	0.235

### Construction of a nomogram based on risk scores and clinical data

**Figure 4:**
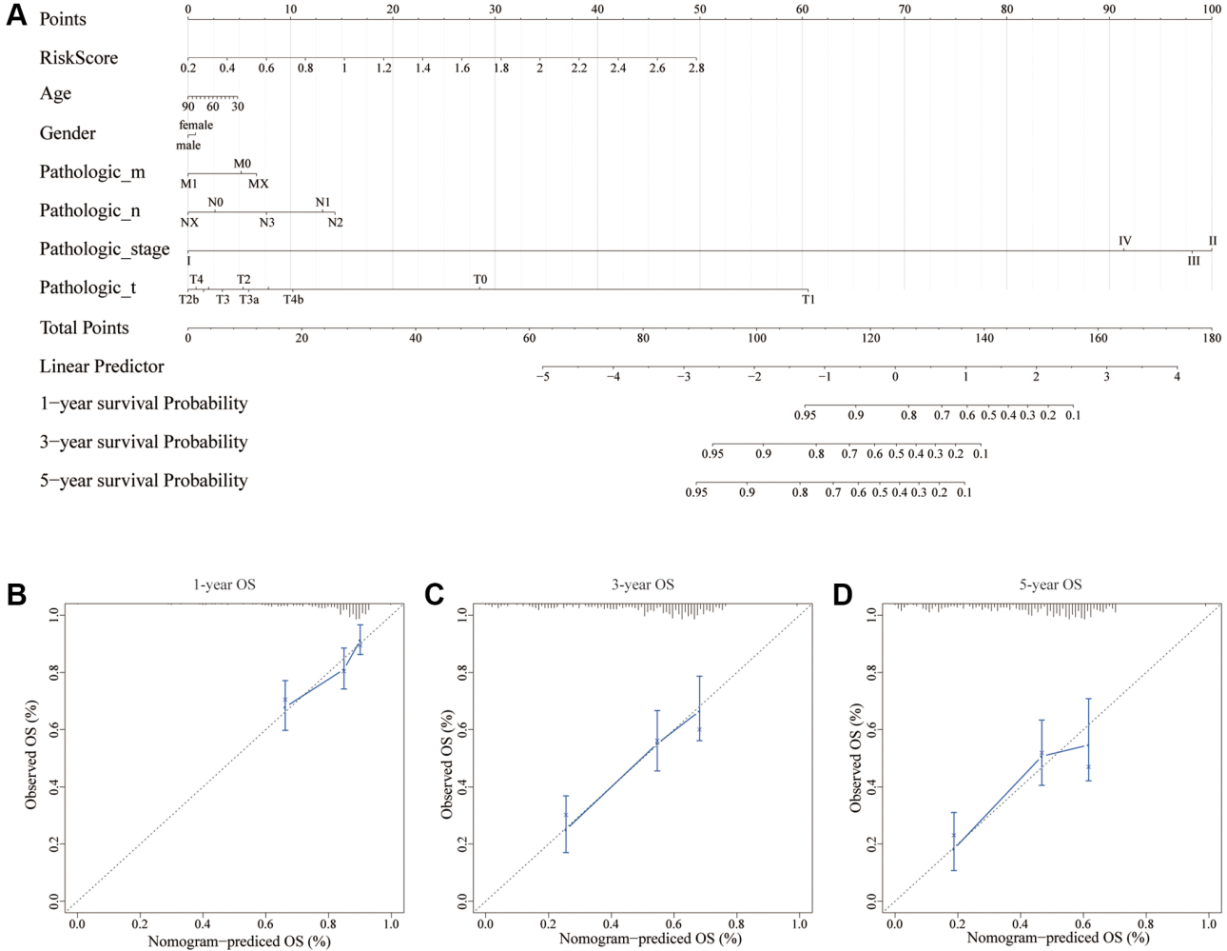
**A nomogram for predicting recurrence in BC.** (**A**) Nomogram based on the signature and clinicopathological features. (**B**) Calibration plot showing that nomogram-predicted 1-year recurrence probabilities corresponded to the actual observed 1-year recurrence probabilities; (**C**) Calibration plot showing that nomogram-predicted 3-year recurrence probabilities corresponded to the actual observed 3-year recurrence probabilities; (**D**) Calibration plot showing that nomogram-predicted 5-year recurrence probabilities corresponded to the actual observed 5-year recurrence probabilities.

Using multiple risk factors, a nomogram can quantitatively assess individual risks in the clinical setting [18, 19]. We constructed a nomogram to predict OS in bladder cancer patients based on factors such as risk score, age, gender, pathological stage, pathological stage, lymph node metastasis, and distant metastasis of bladder cancer patients. As shown in Figure 4A, the above factors are scored according to their contribution to OS, and the 1-year, 3-year and 5-year OS probabilities of bladder cancer patients can be predicted by calculating the sum of the corresponding scores of the above factors. Calibration curves showed a substantial match between actual OS and expected OS, regardless of 1-year follow-up, 3-year follow-up, or 5-year follow-up (Figure 4B–4D).

### Exploring the underlying mechanisms of different risk groups based on gene mutation and differential expression

We further explored differences in gene mutation and expression among different risk groups.

**Figure 5:**
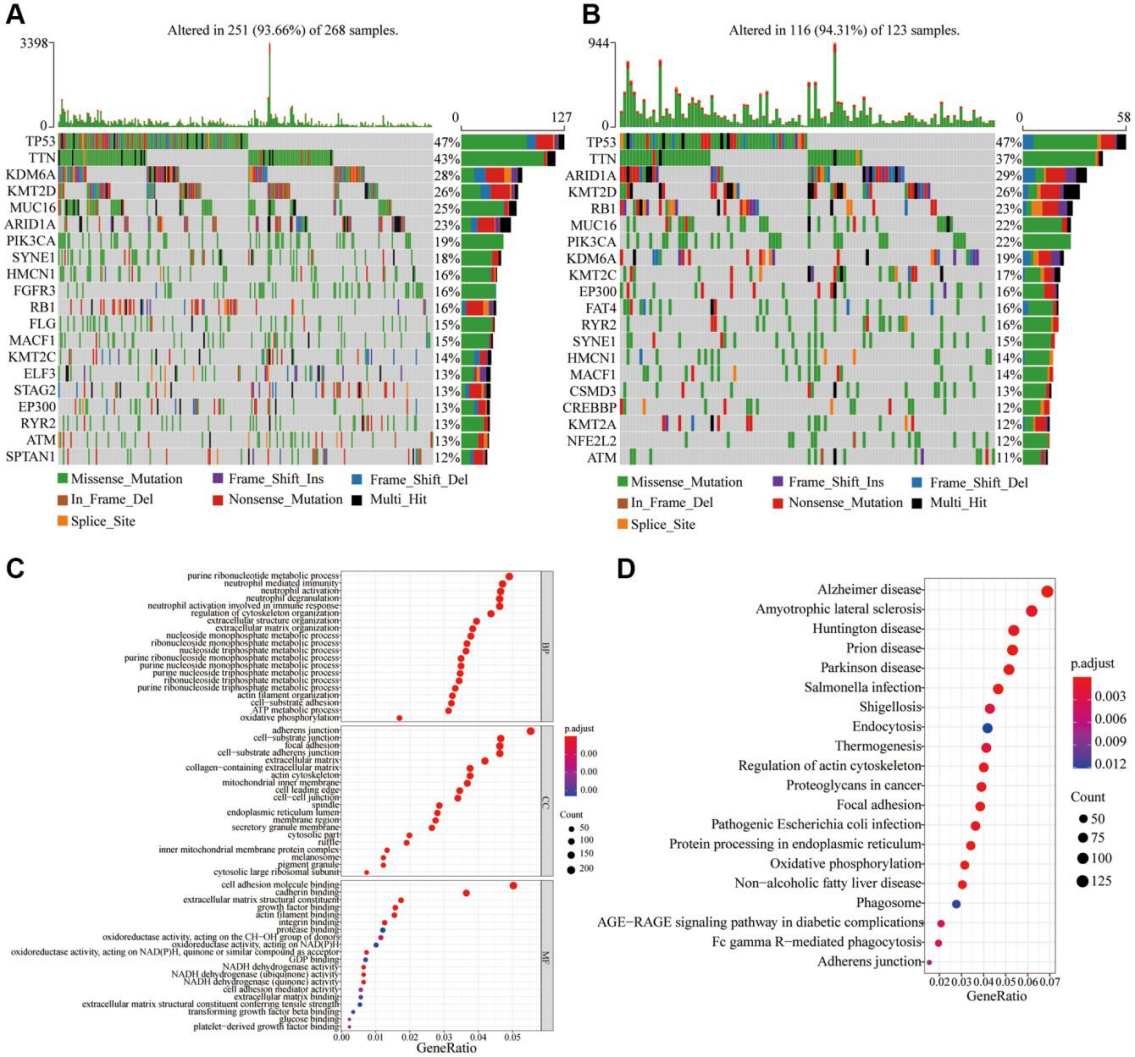
**Frequency of gene mutations and differential expression of genes in different risk groups.** (**A**) Visualization of gene mutations in low-risk score groups; (**B**) Visualization of gene mutations in high-risk score groups; (**C**) GO analysis of differential expression genes in different risk groups; (**D**) KEGG analysis of differential expression genes in different risk groups.

After analyzing the gene mutations, it was found that the overall frequencies of gene mutations in the low-risk and high-risk groups were 93.66% and 94.31%, respectively (Figure 5A, 5B). However, the frequencies of different gene mutations were different. As shown in Figure 5A, 5B, in the low-risk group, the top five gene mutation frequencies were TP53 (47%), TTN (43%), KDM6A (28%), KMT2D (26%), and MUC16 (25%), whereas the top five gene mutation frequencies were TP53 (47%), TTN (37%), ARID1A (29%), KMT2D (26%) and RB1 (23%) in the high-risk group. In the high-risk group, the mutation frequency of ARID1A (29%) and RB1 (23%) was higher than that of the low-risk group (23% and 16%, respectively) (Figure 5A, 5B).

There were 4,025 differentially expressed genes found after the differential analysis of genes in the different risk groups. GO enrichment and KEGG analysis were performed on the differentially expressed genes. As can be seen from the Figure 5C, the biological processes that differentially expressed genes mainly participate in, are neutrophil-mediated immunity, nucleoside metabolic process, and oxidative phosphorylation. Differentially expressed genes mainly constitute cell components such as Adherens junction, Cell-substrate junction, and Focal adhesion. The molecular functions of differentially expressed genes are mainly cell adhesion, oxidoreductase activity, acting on NAD(P)H, and NADH dehydrogenase activity. The KEGG analysis revealed that the main signal pathways involved in differential genes include processes such as infection, Focal adhesion, Oxidative phosphorylation, protein processing in endoplasmic reticulum, Fc gamma R-mediated phagocytosis, and Adherens junction (Figure 5D).

### Analysis of immune cell infiltration in different risk groups

**Figure 6:**
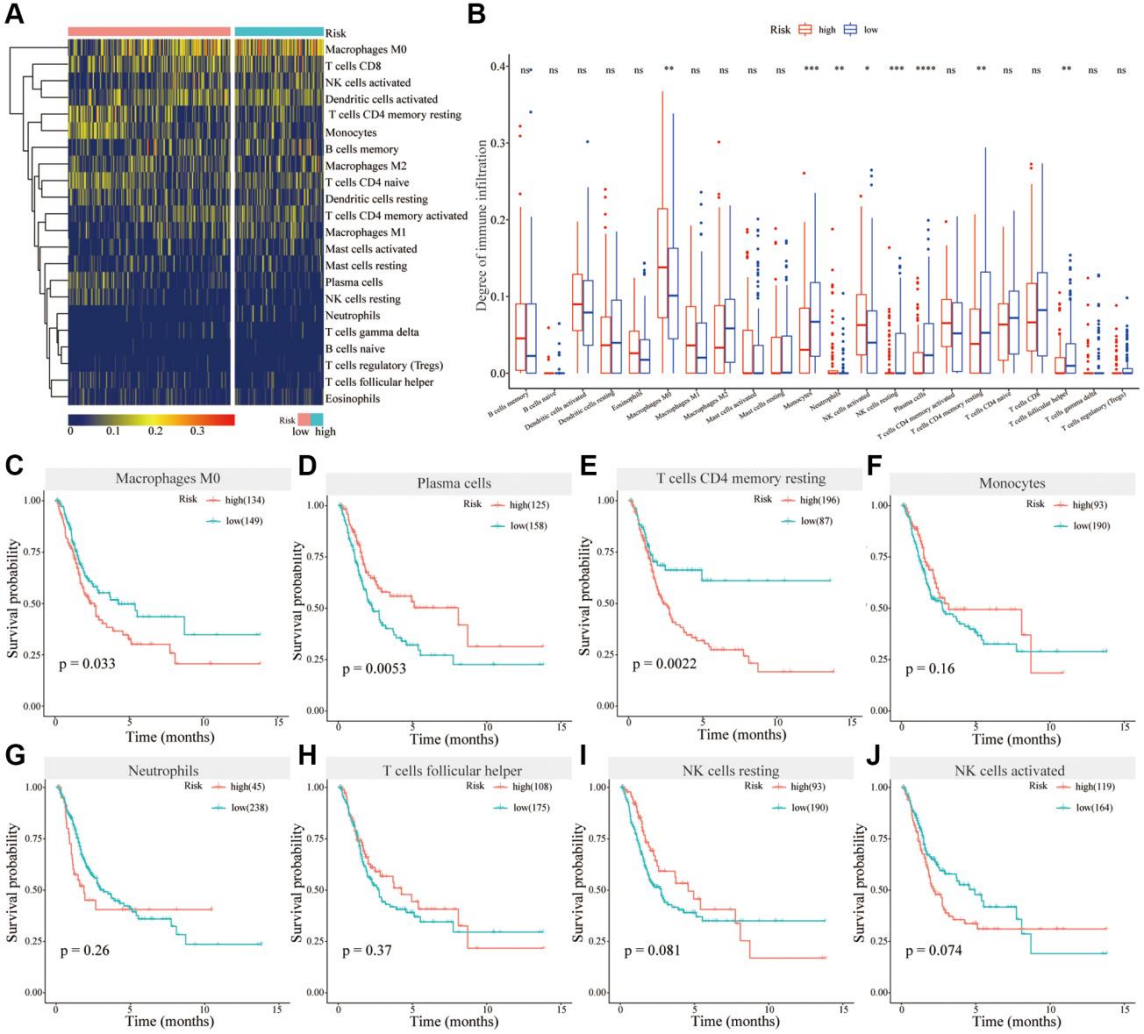
**Analysis of immune cell infiltrations in different risk groups.** (**A**) Heatmap showing immune cell infiltration in different risk groups; (**B**) Differential analysis of immune cells in different risk groups; (**C**–**J**) Survival analysis of Macrophages M0 (**C**), Plasma cells (**D**), T cells CD4 memory resting (**E**), Monocytes (**F**), Neutrophils (**G**), T cells follicular helper (**H**), NK cells resting (**I**) and NK cells activated (**J**) in BC.

Immune infiltration analysis revealed that in the high-risk group, the degree of immune infiltration of Monocytes, plasma cells, NK cells resting, T cells CD4 memory resting, and T cells follicular helper was significantly lower than that of the low-risk group, whereas the degree of immune infiltration of Macrophages M0, Neutrophils, and NK cells activated was significantly higher compared to the low-risk group (Figure 6A, 6B). Survival analysis showed that Macrophages M0, the high levels of infiltrating immune cells in the high-risk group, was associated with the poor prognosis of bladder cancer patients (Figure 6C). Plasma cells, the low-level infiltrating immune cells in the high-risk group, were associated with better prognosis (Figure 6D). The degree of infiltration of T cells CD4 memory resting was higher in the low-risk group than that in the high-risk group, but its high expression was related to the poor prognosis for BC patients (Figure 6E). The remaining differentially infiltrated immune cells were not associated with the prognosis of bladder cancer patients (Figure 6F–6J). Therefore, different levels of immune cell infiltration are closely associated with the prognosis of bladder cancer patients in high-risk and low-risk groups.

### Validation of differential expression of 11 FRGs

**Figure 7:**
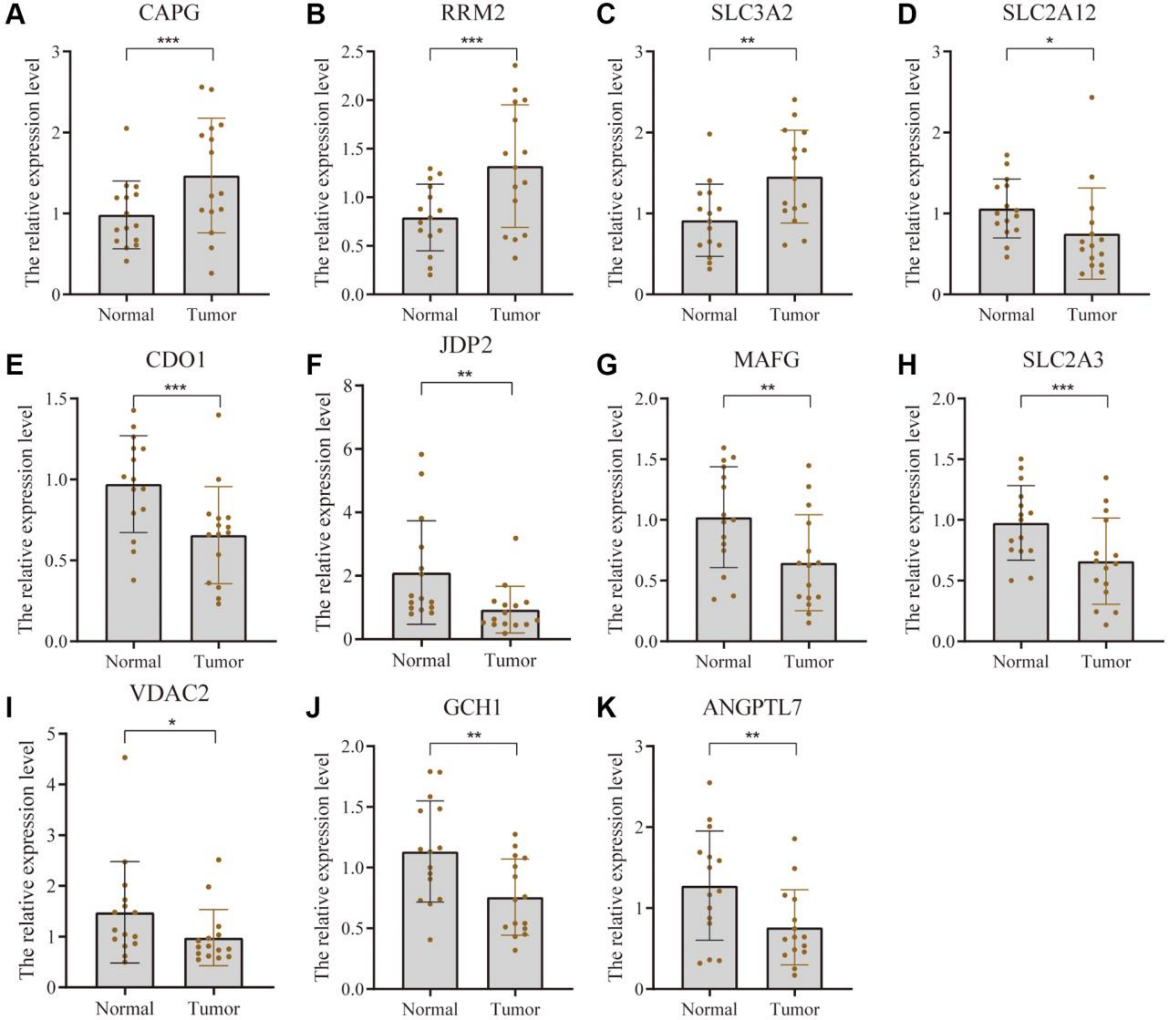
**Validation of differential expression of 11 FRGs in tissues using PCR.** Bar graphs show differential expression of CAPG (**A**), RRM2 (**B**), and SLC3A2 (**C**) was higher whereas that of SLC2A12 (**D**), CDO1 (**E**), JDP2 (**F**), MAFG (**G**), SLC2A3 (**H**), VDAC2 (**I**), GCH1 (**J**), and ANGPTL7 (**K**). ^*^ represents *p*-value less than 0.05, ^**^ represents *p*-value less than 0.01, and ^***^ represents *p*-value less than 0.001.

The expression of 11 FRGs was verified using tumor tissues and para-cancerous tissues of 15 pairs of bladder cancer patients. Figure 7 shows that the expression level of CAPG, RRM2, and SLC3A2 was higher whereas that of SLC2A12, CDO1, JDP2, MAFG, SLC2A3, VDAC2, GCH1, and ANGPTL7 was lower in bladder cancer tissue compared with the adjacent tissue.

**Figure 8:**
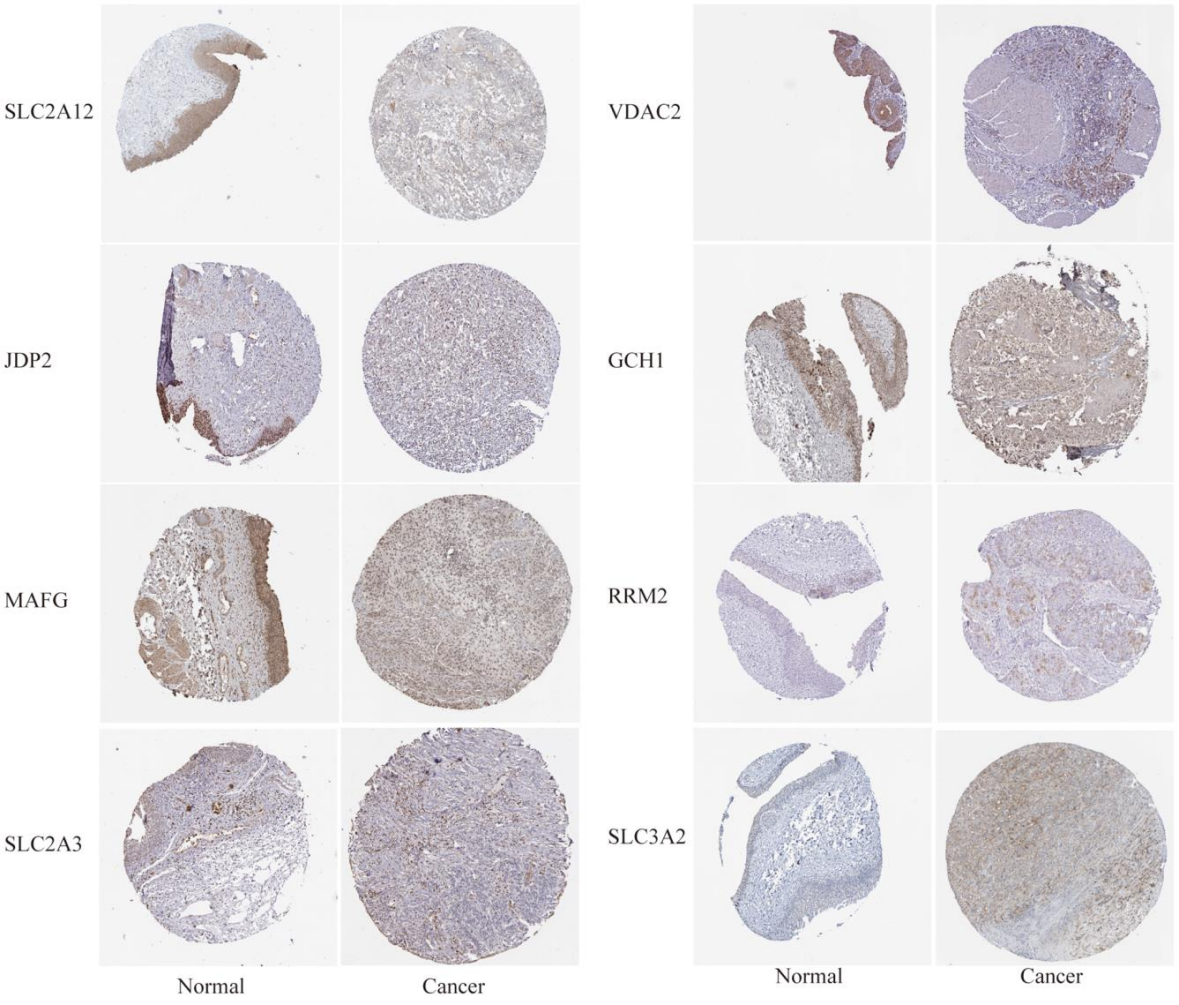
The differential expression of some FRGs at the protein level was verified using the human protein atlas (HPA, https://www.proteinatlas.org/) website.

In addition, we determined the expression levels of FRG proteins using HPA tool. It can be seen from Figure 8 that compared with normal tissues, the expression level of SLC2A12, JDP2, MAFG, SLC2A3, VDAC2, and GCH1 protein was lower in bladder cancer whereas that of RRM2 and SLC3A2 proteins was higher in BC tissues.

## DISCUSSION

Bladder cancer, mainly urothelial carcinoma, is one of the most common solid tumors, and its incidence is higher in men than in women [20]. About 75% of newly diagnosed patients have NMIBC, and the remainder are MIBC or metastatic bladder cancer. In general, the 5-year survival rate for NMIBC is 90%, but that of MIBC and metastatic bladder cancer drop dramatically to less than 50% and less than 15%, respectively [21, 22]. Because bladder patients lack prognostic targets to predict the prognosis of patients, there is an urgent need to find a gene set that can effectively predict the prognosis of BC.

In 2012, Dixon first proposed ferroptosis, which is an iron and reactive oxygen species-dependent programmed cell death that is distinct from other cell deaths [23]. Its main characteristics include the reduction or disappearance of mitochondrial cristae, the rupture of mitochondrial outer membrane and the condensation of mitochondrial membrane [24, 25]. Mitochondria undergo characteristic changes after ferroptosis induction, and the abnormal changes are due to the loss of biological function caused by lipid peroxidation of the mitochondrial membrane [26, 27]. Ferroptosis regulates the progression of a variety of tumors, such as colorectal cancer, gastric cancer, ovarian cancer, prostate cancer, breast cancer, lung cancer and melanoma [24, 28]. For example, knockdown of Nedd4 inhibits VDAC2/3 protein degradation, thereby increasing the sensitivity of melanoma cells to the ferroptosis inducer erastin [28]. The sensitivity of different types of cancer to ferroptosis was significantly different. Compared with other cancer cells, erastin is more likely to cause ferroptosis in diffuse large B-cell lymphoma and renal cell carcinoma [29]. Ferroptosis regulators play an important role in the treatment of tumors. A variety of chemotherapeutic drugs combined with ferroptosis inducer erastin significantly improved the antitumor activity of chemotherapeutic drugs [30]; the prognosis is better than traditional chemotherapy alone [24]. The rapid development of ferroptosis in cancer provides prospects for its application in cancer treatment. However, there are very few studies on ferroptosis in BC. Therefore, it is particularly important to find FRGs related to the prognosis of BC, and to explain the mechanism of FRG genes in the development of BC.

Differential analysis of data from the TCGA cohort was performed to identify reliable prognostic biomarkers for BC. In total, 4953 differential genes were screened, of which 80 were differentially expressed FRG genes. After prognostic analysis of the differentially expressed FRG, 37 FRG genes were found to be related to the prognosis; 25 were down-regulated and 12 were up-regulated. Given that PLIN4 and TAZ are not in the GSE13507 dataset, the LASSO regression method was used to construct a prognostic model in the TCGA-training set based on the remaining 35 genes. At last, 11 prognostic FRGs (SLC2A12, CDO1, JDP2, MAFG, CAPG, RRM2, SLC2A3, SLC3A2, VDAC2, GCH1, and ANGPTL7) were finally identified from the 35 genes by L1-penalized Cox proportional hazards regression. They were used to effectively construct a prognostic model which was validated on the TCGA-testing set and GSE13507 cohort. These FRG can be used as clinically valuable biomarkers. The overall survival of BC patients can be divided into two different risk groups based on the prognostic characteristics of 11 FRG. Compared with the low-risk group, the prognosis of the high-risk group is significantly lower according to the TCGA and GSE13507. Multivariate Cox proportional hazards analysis showed that the FRG-based risk score in the TCGA cohort was an independent prognostic factor, whereas other clinical data were not. This predictive model predicts the prognosis of bladder cancer patients based on the expression levels of 11 genes, which is more economical and more clinically feasible than high-throughput sequencing. The limitation of this model is that its accuracy is not ideal for predicting the survival of BC patients. However, compared with models established in other studies, the performance of our model based on 11 FRG genes is acceptable [31]. It can be used to predict the prognosis of bladder cancer patients.

To study the potential molecular mechanism of gene signatures on prognosis, BC patients were divided into high-risk and low-risk groups according to the risk score. Gene mutations and differential expression between different risk groups were studied. Studies have shown that the overall frequency of gene mutations in different risk groups is the same, but the frequency of different gene mutations varies. In the high-risk group, the mutation frequency of ARID1A and RB1 was higher than that in the low-risk group. In tumors, knockout of ARID1A impaired transcriptional activation of SLC7A11 and makes tumor cells more sensitive to GSH metabolic pathway inhibitors due to insufficient cysteine supply [32]. The loss of ARID1A expression has been associated with poor prognosis, and its mutations confer bladder cancer non-stem cells the self-renewal [33, 34]. Thus, in the high-risk group, patients may be susceptible to ferroptosis due to ARID1A mutations. Retinoblastoma 1 (RB1) are related to the carcinogenic effects, whose deletion is a biomarker of poor prognosis in MIBC [35]. Mutations in the RB1 gene are associated with significantly reduced disease-specific survival rates [36]. Simultaneous knockdown of TP53 and RB1 can promote the lineage transformation of urothelial cancer cells to neuroendocrine like tumor cells, and reduce the response to targeted drugs [37]. But the relationship between RB1 and ferroptosis is still unclear, and there is clinical value for further research. The difference in mutation frequency of different genes may be the potential mechanism for differences in prognosis among different risk groups. Also, GO enrichment and KEGG analysis were used following differential gene expression. Comprehensive GO analysis and KEGG analysis showed that differentially expressed genes were associated with immune regulation, cell adhesion and oxidative phosphorylation. During oxidative phosphorylation involving iron in mitochondria, cells produce reactive oxygen species (ROS) and forms ATP. ROS levels that exceed cellular antioxidant capacity lead to oxidative stress and induce ferroptosis [30]. Recent studies suggested that cell–cell contact can prevent erastin-induced ferroptosis, and intercellular contacts, especially adherens junctions, can also control ferroptosis [38]. Interestingly, ferroptosis has been found to participate in immune regulation. Some studies reported that ferroptosis affected immune cells in two fundamentally different ways. It also influences the number and function of immune cells themselves. Ferroptosis cells can be recognized by immune cells which triggers a series of inflammatory or specific responses [39]. We further analyzed the degree of immune infiltration in different risk groups of bladder cancer patients. The results showed that the degree of infiltration of macrophages M0 was significantly increased in the high-risk group contributing to poor prognosis of BC patients. In the high-risk group, low-level infiltrating immune cells, such as plasma cells, are associated with a better prognosis in patients with BC. Therefore, we speculate that in the high-risk group, macrophages are ferroptotic-resistant or ferroptosis inhibition increases the number or function of macrophages, and the opposite is true for plasma cells. Deregulation of tumor immune cell infiltration may explain prognostic differences between patients in different risk groups.

This model may be a useful classification tool for the prognosis and diagnosis of BC. In practice, it may be more conventional and more cost effective, because this prognostic model can reduce the need for whole genome sequencing in patients with bladder cancer. The nomogram, which combines our signature with traditional clinical parameters, shows a significantly improved performance, indicating that it more accurately reflects the huge heterogeneity of BC.

However, there were some limitations to this study. The study was mainly based on data from the TCGA cohort, and most of the patients were white or Asian. Extra care should be taken in the extrapolation of our findings to patients from other races. The bioinformatics analysis in our study is descriptive, and further functional experiments are needed to clarify the potential mechanism of these genes.

## CONCLUSIONS

In summary, a prognostic 11 FRG gene signature was established based on the TCGA and GEO BC cohort. Risk scores based on 11 FRGs are independent predictors of prognosis in BC patients. The nomogram based on genetic characteristics and clinicopathological characteristics could accurately predict the 1-year, 3-year, and 5-year survival rates of BC patients. It can thus be used to guide individualized treatment and medical decision making.

## Supplementary Materials

Supplementary Figure 1

Supplementary Table 1

Supplementary Table 2

Supplementary Table 3

Supplementary Table 4
