# Supplementary material for: Development and validation of a novel model for predicting the survival of bladder cancer based on ferroptosis-related genes
Source: Aging (Albany NY). 2022 Nov 17;14(22):9037–55. doi: 10.18632/aging.204385 (PMC9740359; doi:10.18632/aging.204385)
Supplement: Supplementary Figure 1 [file aging-14-204385-s001.pdf]

SUPPLEMENTARY FIGURE

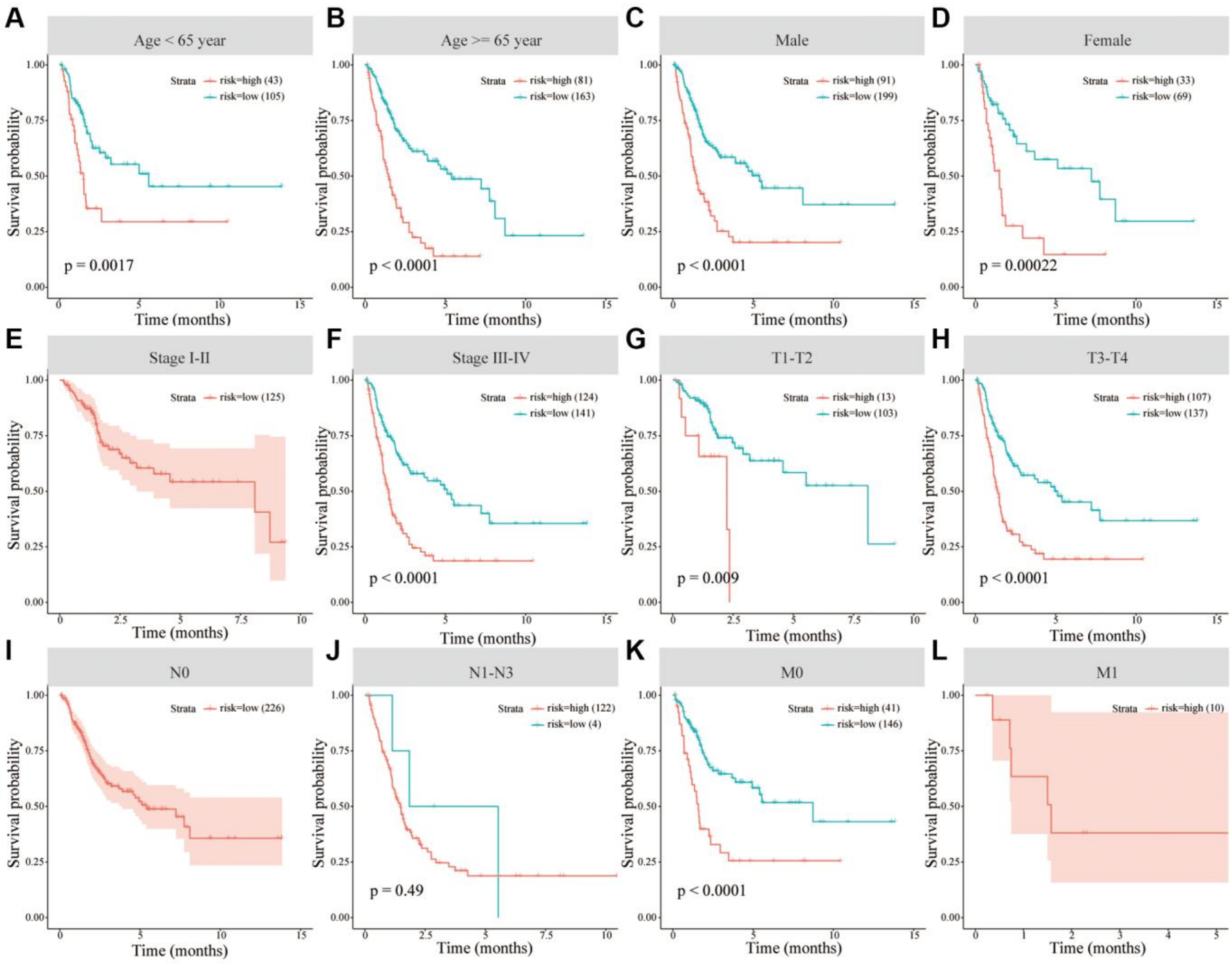

Supplementary Figure 1. The prognostic value of risk scores in different clinical groups, such as Age <65 year (**A**), Age ≥ 65 year (**B**), Male (**C**), Female (**D**), Stage I–II (**E**), Stage III–IV (**F**), pathological stage T1-T2 (**G**), pathological stage T3-T4 (**H**), none lymph node metastasis (**I**), lymph node metastasis N1-N3 (**J**), none distant metastasis (**K**), distant metastasis M1 (**L**).
