# Supplementary material for: Development and validation of a novel model for predicting the survival of bladder cancer based on ferroptosis-related genes
Source: Aging (Albany NY). 2022 Nov 17;14(22):9037–55. doi: 10.18632/aging.204385 (PMC9740359; doi:10.18632/aging.204385)
Supplement: Supplementary Table 1 [file aging-14-204385-s002.pdf]

SUPPLEMENTARY TABLE

Supplementary Table 1. PCR primer sequences.

Genes	Forward (5′–>3′)	Reverse (5′–>3′)
SLC2A12	CATTAGCACCATCCCTGCCA	TGGTCCAAAGACACCCTCCT
CDO1	ATACGGAACCTGCTGTGAGC	GCACGTGGTAGGTAGCCTTT
JDP2	CCGATGCCGGAACAAGAAGA	TGGCTGTGCTGAACCTCATT
MAFG	TCTAGGGCTTGGGCTGATCT	TGTAGCCCTTGTCTGCACTG
CAPG	AGGAAAGTAGGAGTGTTGAAAGA	AAGACGCCCTGGTTCTCTTG
RRM2	GCCACACCATGAATTGTCCG	ATGGTAAGTCACAGCCAGCC
SLC2A3	TGTAGCCTTCTTTGAAATTGGACC	AGCAGCATTCAGAAGCGTCC
SLC3A2	GGGACTAACTCCTCCGACCT	GGCCAATCTCATCCCCGTAG
VDAC2	CAACTGCACTCGTTTTGGCA	GCTGCTGGTCACAATGGAAA
GCH1	CGGGAGGATCCAAAGACTCG	ACCGGACAGACAGACAATGC
ANGPTL7	GGTATGGCTGGCATGGATCT	TCCAGTCTCCACGTGTCTCA
GAPDH	GAGTCAACGGATTTGGTCGT	TTGATTTTGGAGGGATCTCG
